# Effect of recombinant human growth hormone therapy on blood lipid and carotid intima-media thickness in children with growth hormone deficiency

**DOI:** 10.1038/pr.2017.271

**Published:** 2018-01-17

**Authors:** Ming Chen, Dongmei Gan, Yili Luo, Sharvan Rampersad, Lu Xu, Shaoling Yang, Nan Li, Hong Li

**Affiliations:** 1 Department of Endocrinology, Shanghai Tenth People’s Hospital, Shanghai, China; 2 Department of Endocrinology, Ningbo Women and Children’s Hospital, Ningbo, China

## Abstract

**Background:**

Reports on the association between growth hormone deficiency (GHD) and cardiovascular risk factors in children are limited. We aim to investigate the effect of different doses of recombinant human growth hormone (rhGH) therapy on blood lipid and carotid intima-media thickness (cIMT) in Chinese GHD children.

**Methods:**

Ninety children, including sixty isolated GHD children and thirty healthy children, were enrolled. GHD children were randomly divided into two groups (A and B) according to the rhGH dose given: group A received 0.23 mg/kg/week and group B received 0.35 mg/kg/week for 12 months. The TC, TG, LDL-C, HDL-C, and cIMT at baseline and after treatment were measured.

**Results:**

The height, weight, and height velocity improved significantly over 12 months of rhGH therapy in all GHD children. At baseline, GHD children in both the treatment groups showed significantly higher total cholesterol (TC), triglyceride (TG), low-density lipoprotein-cholesterol (LDL-C), cIMT, and lower high-density lipoprotein-cholesterol (HDL-C) than healthy children (all *P*≤0.033). After the 12-month rhGH therapy, a significant decrease in the TC, TG, LDL-C, and cIMT, as well as a significant increase in the HDL-C (*P*≤0.046), was observed in the GHD children, with change in the group B being even more marked.

**Conclusions:**

The RhGH replacement therapy in GHD children can improve both the blood lipid profile and carotid intima-media thickness, with higher-dose rhGH therapy showing superior effects.

Growth hormone deficiency (GHD) is an endocrine disease that can affect an individual’s life during childhood, adolescence, and adulthood. Recombinant human growth hormone (rhGH) replacement therapy is a standard treatment option for normalizing the final adult height in GHD children. Growth hormone (GH) can promote linear growth, accelerate protein synthesis, and stimulate bone growth; moreover, it has specific effects on the body composition, muscle strength, bone density, blood lipid level, and endothelial function (1, 2).

Dyslipidemia in childhood is a risk factor for adult cardiovascular diseases, such as arteriosclerosis. Adults and adolescents with severe GHD also frequently exhibit dyslipidemia, abdominal obesity, insulin resistance, hypertension, increased carotid intima-media thickness (cIMT), and changes in cardiac structure and function (3, 4). cIMT is a non-invasive predictive indicator of atherosclerotic processes in the coronary arteries. Previous research has shown that both short-term and long-term rhGH therapies have specific effects on abdominal fat, blood lipids, and intimal thickness regardless of age in adults or children (5, 6, 7).

Few data are available regarding the blood lipid and cIMT levels as well as the variations in these parameters following rhGH therapy in GHD children from Asian, particularly those from Chinese populations. In addition, the dose of rhGH for treating GHD children, as recommended by the *Diagnosis and Treatment Guidelines for Chinese Short Stature Children*, is 0.23–0.35 mg/kg/week, which can promote linear growth in a dose-dependent manner (8). However, which dose of rhGH is more effective in improving blood lipid and cIMT in GHD children remains unknown. Therefore, this study investigated the effect of different doses of rhGH therapy on blood lipid and cIMT levels in Chinese GHD children.

## Methods

### Subjects

This was a prospective case–control study. In total, 60 cases of isolated GHD children aged 5–10 years (37 boys and 23 girls) who were consulted and treated at the Ningbo Women and Children’s Hospital (Zhejiang, China) between February 2014 and April 2015 were enrolled in the treatment group. The inclusion criteria were as follows: (i) age 5–10 years; (ii) Tanner 1; (iii) short stature, defined as a height more than two SDs below the population mean; (iv) bone age <2 SD; (v) height velocity (HV) <5cm/year; and (vi) two different GH provocative tests indicating a GH peak <10 μg/l (<5 μg/l: complete GHD, 5–9.9 μg/l: partial GHD). The exclusion criteria were as follows: (i) any acute severe illness during the previous 1 year; (ii) any cardiovascular, respiratory, renal, liver, or endocrine disease; (iii) family or personal history of lipid disorders; (iv) multiple pituitary hormone deficiency; (v) nutrient deficiency; and (vi) the use of hormonal drugs or drugs that affected blood lipid during the previous 1 year. All participants were blinded to the treatment type and randomized to group A (mean age: 7.53 years, 19 boys and 11 girls) or group B (mean age: 7.68 years, 18 boys and 12 girls), with 30 cases in each group.

The control group included 30 healthy age- and sex-matched children (mean age: 7.55 years, 18 boys and 12 girls) in the same pubertal stage, and any organic diseases were excluded based on physical examinations in our hospital. Controls were selected among children referred to our outpatient clinic for health examinations.

This study was performed with parental consent and with the approval of the hospital’s ethics committee.

### Diagnostic Methods

Anthropometry, laboratory tests, and radiological examination were performed prospectively at baseline and after 12 months of therapy.

A human height meter (Suhong, Jiangsu, China) and a body-weighing scale (Suhong) were used to measure height and weight, with an accuracy of 0.1 cm and 0.1 kg, respectively. Each subject removed their shoes and stood on the baseboard in the attention position, with both upper limbs hanging down naturally and heels together. The subject was asked to stand such that the heels, sacrococcygeal region, and bilateral scapular areas were placed in contact with the column; the trunk was straight and upright; and the head was in the normal position. All subjects were measured twice in order to calculate a mean. BMI (the weight in kilograms divided by the square of the height in meters) and SD score (SDS) for all anthropometric parameters were based on published normative data for Chinese children (9, 10).

Examinations such as routine tests for blood, urine hepatorenal function, electrolyte, blood lipid, insulin-like growth factor-1 (IGF-1), IGF-binding protein 3 (IGFBP-3), thyroid function, fasting plasma glucose (FPG), bone age; carotid artery ultrasound; and pituitary magnetic resonance imaging were conducted. In girls, a chromosomal examination was also conducted. We also performed two GH provocative tests (using arginine and clonidine) and recorded the birth history, past medical history, growth and development history, genetic history, and dietary habits. Pubertal stage was assessed according to the method of Tanner and Whitehouse (11). A diagnosis of GHD was considered based on the medical history, physical examination, and blood tests, as described above.

### Treatment and Diagnostic Criteria

GHD children were given a subcutaneous injection of rhGH (10 mg/tube; Changchun Kinsey Pharmaceutical Industry, Jilin, China) every day before sleeping, among whom group A received rhGH 0.23 mg/kg/week and group B received rhGH 0.35 mg/kg/week. The injection sites were the upper arm, thigh, or abdomen; injection in the same site was avoided in the short term and the treatment duration was 12 months. Growth and developmental indices were examined regularly. Thyroid function, serum triglycerides (TG), total cholesterol (TC), low-density lipoprotein-cholesterol (LDL-C), high-density lipoprotein-cholesterol (HDL-C), and cIMT were assessed before and after treatment. The diagnostic criteria were as follows (12): hyperlipidemia: TC≥5.18 mmol/l, LDL-C≥3.37 mmol/l, or TG≥1.70 mmol/l; low HDL-C level: HDL-C≤1.04 mmol/l; the critical values: TC: 4.40–5.15 mmol/l and LDL-C: 2.85-3.34 mmol/l; the normal values: TC<4.40 mmol/l and LDL-C<2.85 mmol/l.

### Detection Methods

(i) Bone age was evaluated using plain X-ray of the left hand and wrist according to the standards of Greulich and Pyle (13). (ii) Blood lipid inspection was carried out where all subjects were required to fast for 10–12 h and 1 ml of fasting venous blood (non-anticoagulant) was collected the following morning. The serum TG, TC, LDL-C, and HDL-C levels were determined immediately using a fully automatic analyzer (AU5800, Beckman, Coulter Inc, Brea, CA, USA); IGF-1 and IGFBP-3 were measured using solid-phase immunoradiometric assay. (iii) cIMT was detected using a diagnostic ultrasound system, with the probe set at 12 MHz. The affected children were in the supine position, in which their heads deviated to the non-inspection site and a slightly higher pillow was placed at the back of the neck. Bifurcation of the common carotid artery was sounded in order to determine the cIMT. Data were recorded by two investigators who were blinded to the subject’s status.

### Statistical Analysis

SPSS 19.0 software (Windows Edition) was used for all statistical analyses. Results are expressed as mean±SD unless specified otherwise. Between-group comparisons before treatment were performed using univariate linear model analysis, whereas comparisons after treatment were performed using repeated linear model analysis, adjusting for age, sex, and BMI. Differences between continuous variables including TC, TG, LDL-C, HDL-C, cIMT, IGF-1, IGFBP-3, and FPG in the GHD groups (before and after therapy for 12 months) were analyzed using the paired *t*-test. Significant differences were accepted at *P*<0.05.

## Results

### Comparison of Height, Weight, and HV Between Group A and Group B

No patients were lost to follow-up. At baseline, no significant differences were found in terms of height, weight, and HV between groups A and B. After 12 months of rhGH therapy, GHD children exhibited significant increases in height (*P*_A_=0.012, *P*_B_=0.025), weight (*P*_A_=0.023, *P*_B_=0.029), BMI (*P*_A_=0.049, *P*_B_=0.006), and HV (*P*_A_=0.033, *P*_B_=0.024). After converting the height, weight, and BMI into SDSs, GHD patients still showed significant increases in height SDS (*P*_A_<0.001, *P*_B_<0.001) and weight SDS (*P*_A_=0.016, *P*_B_=0.009), but not in BMI SDS (*P*_A_=0.689, *P*_B_=0.551). Moreover, HV was notably higher in group B than in group A (Table 1).

### Comparison of Blood Lipid Levels and cIMT Across the Three Groups

At baseline, GHD children in both treatment groups exhibited profound impairment in their lipid profiles, including higher TC (group A/group B vs. controls, *P*=0.016/0.018), TG (*P*=0.022/0.027), and LDL-C (*P*=0.001/0.009), and lower HDL-C (*P*=0.012/0.009), IGF-1 (*P*=0.001/0.001), and IGFBP-3 (*P*=0.031/0.022), as well as significant increases in cIMT (*P*=0.029/0.032) compared with controls. The values were similar for group A and group B.

**Figure 1:**
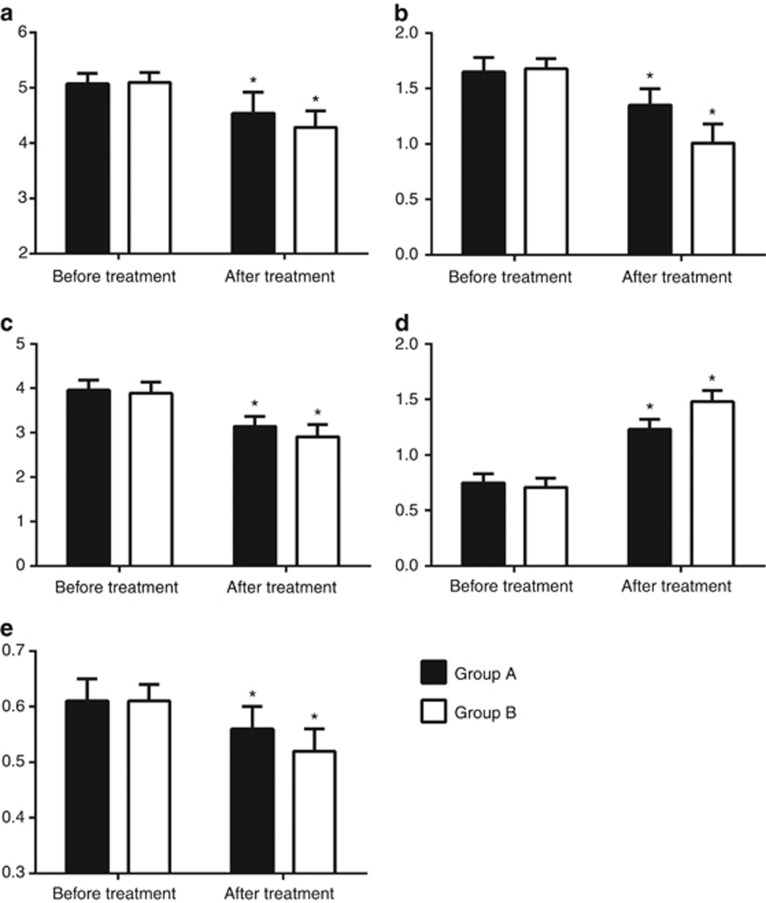
Comparison of blood lipid level and cIMT in treatment groups before and after treatment. (**a**) TC (mmol/l), (**b**) TG (mmol/l), (**c**) LDL-C (mmol/l), (**d**) HDL-C (mmol/l), (**e**) cIMT (mmol/l). *P<0.05 compared with the data before treatment. cIMT, carotid intima-media thickness; HDL-C, high-density lipoprotein-cholesterol; LDL-C, low-density lipoprotein-cholesterol; TC, total cholesterol; TG, triglyceride.

After 12 months of rhGH therapy, we observed significant reductions in TC (*P*_A_=0.033, *P*_B_=0.038), TG (*P*_A_=0.046, *P*_B_=0.031), and LDL-C (*P*_A_=0.045, *P*_B_=0.035), and significant increases in HDL-C (*P*_A_=0.032, *P*_B_=0.026), IGF-1 (*P*_A_=0.025, *P*_B_=0.010), IGFBP-3 (*P*_A_=0.028, *P*_B_=0.015), the changes being more distinct in group B. Moreover, cIMT (*P*_A_=0.043, *P*_B_=0.039) decreased significantly in the GHD children, among whom the reduction was more obvious in group B, but the cIMT was still more than that in the control group. With the exception of TC, IGF-1, and IGFBP-3, all of the values differed greatly after the treatment between group A and group B (Figure 1 and Table 2).

### Adverse Reactions after Treatment

Reduced levels of T4 were evident in both groups during treatment; however, they were improved after the administration of thyroxine tablets. Increased levels of FPG were detected in both treatment groups, but they returned to normal after the suspension of treatment. However, FPG did not increase further following re-commencement of the treatment, although two cases in group A developed lower limb edema, which was self-limiting without any special treatment, and two cases in group B developed knee pain, which was relieved after calcium supplement therapy. The occurrence rates for adverse reactions in group A and group B were 26.67% and 33.33%, respectively; however, the difference between the groups was not statistically significant (*χ*^2^=0.317, *P*=0.573; Table 3).

## Discussion

RhGH was first produced in 1985, and it provided hope for the treatment of all patients with GHD. After the rapid clinical application of rhGH, its therapeutic effects were verified extensively, and it became the predominant drug for treating GHD (14). The effect of rhGH on lipid metabolism has also been the focus of much attention in recent years where dyslipidemia is closely associated with cardiovascular risk factors. In fact, it has emerged that GHD children are highly likely to have an unfavorable lipid profile (15, 16).

In agreement with previous studies, our results demonstrated that the levels of TC, TG, and LDL-C were higher in GHD children, whereas the HDL levels were lower compared with those in age- and sex-matched healthy children. It is currently considered that GH can promote lipolysis, reduce the lipid content of tissues, and regulate the lipid-dissolving rate of adipocytes by activating the β adrenergic receptor in adipocytes (17, 18). Moreover, GH can upregulate the mRNA expression level of the liver LDL-C receptor, enhance the liver’s capacity for LDL-C uptake, increase blood lipid metabolism, and reduce the rate of LDL-C production, thereby obtaining a notable lipid-lowering effect (19). However, in GHD subjects, there is a reduction in the liver’s lipid-lowering and -dissolving function, which leads to remarkable higher levels of TC and LDL-C in GHD children compared with those in normal children (20). In addition, it has been reported that the lipid metabolism in GHD children is improved greatly after rhGH therapy (15, 21). Efforts are still required to elucidate the other possible mechanisms of dyslipidemia in GHD children.

In the present study, we observed significant reductions in the TC, TG, and LDL-C levels, as well as a significant increase in HDL-C in GHD children following 12 months of rhGH therapy, although the changes were more distinct in the high-dose group. This indicates that the use of higher rhGH doses has greater benefits in terms of the lipid profile. Our findings are in agreement with the results reported by Van *et al.* (22), who demonstrated a long-term beneficial effect of GH therapy on the HDL-C, LDL-C, and free fatty-acid levels in 59 GHD children after GH replacement therapy for 6 years. More recently, De Marco *et al.* (7) found alterations in the lipid profiles of GHD children, which were characterized by increases in TC and LDL-C; however, these alterations were reversible after GH treatment for 12 months. In addition, a beneficial effect of GH therapy on lipid metabolism has been documented in several studies (23, 24, 25), whereas the discontinuation of rhGH therapy after the final height has been achieved contributes to an increase in unfavorable lipid profiles (3, 26, 27).

According to recent research, GHD may increase the risk of morbidity and mortality due to cardiovascular disease in adults (2, 28). The beneficial effects of GH on lipid metabolism as well as the body composition and atherogenic risk have been noted in GHD children (29). In general, GHD children have an increased BMI, increased body lipid content, and increased blood lipids, and these factors are extremely likely to induce cardiovascular diseases such as atherosclerosis after these children reach maturity. CIMT represents the area of tissue from the luminal edge of the artery to the boundary between the media and adventitia, and it is widely recognized as an important indicator for assessing changes in the vascular structure in early atherosclerosis. It is currently considered that atherosclerosis is a disease that develops in childhood with the onset in adulthood. The lipids in the arterial intima start to be deposited in childhood and organ ischemia occurs when the bloodstream flow is reduced to a certain degree; thus, lipid metabolic disorder in childhood will undoubtedly increase the risk of atherosclerosis (30, 31). A recent study compared adults with GHD and controls matched for gender, body weight, and age, and showed that the cIMT was associated with more atheromatous plaques in the femoral and carotid arteries (32). Another study involving 34 GHD patients showed that GHD tends to result in more extensive cIMT thickening in child-onset than adult-onset GHD patients, thereby suggesting that child-onset GHD patients have an increased early arteriosclerotic risk (33).

However, few studies have considered cIMT and the risk of atherosclerosis in GHD children and adolescents. In children, there are fewer influential factors such as smoking, drug administration, and stress than in adults; therefore, the results are more reliable and meaningful. Our study indicated that the mean cIMT was significantly thicker in GHD children than the controls at baseline, and after 12 months of rhGH therapy the cIMT of GHD children had decreased significantly, where the high-dose group exhibited more beneficial effects, although the cIMT was still thicker than that in the control group. Our findings agree with the results reported by Szczepańiska *et al.* (34), who showed that GHD children had significantly elevated cIMT values compared with healthy children, thereby indicating the onset of atheromatosis. Lanes *et al.* (35) also observed a reduction in the cIMT and arterial stiffness in adolescents after GH replacement, as found in the present study. Furthermore, some studies have observed that improvements in atherogenic risk factors were poorer following the withdrawal of GH treatment (36, 37), which supports the beneficial effect of GH therapy on cardiovascular risk. In 2015, the European Society of Paediatric Endocrinology emphasized that GH can improve the blood lipid levels and reduce the carotid intimal thickness, as supported by the results of our study (14). Overall, these results indicate that rhGH therapy has protective effects on cardiovascular features in GHD children.

However, not all studies have obtained the same findings. For example, a prospective study of 23 child-onset GHD adolescents (aged 15–20 years) showed that 6 months of rhGH treatment in adolescents with confirmed GHD did not result in significant changes in the common carotid arteries, whereas in adolescents who were not confirmed as having GHD cIMT increased during rhGH therapy and normalized after 12 months of rhGH withdrawal (38). The direct or indirect effects of rhGH on vascular endothelial cells are not fully understood and additional exploratory studies are required.

The therapeutic process of rhGH is frequently accompanied by some changes. RhGH leads to reduced sensitivity to insulin; therefore, the fasting blood glucose exhibits a transient increase, but it is insufficient to exceed the glucose tolerance threshold. Furthermore, the blood glucose level returns to normal after rhGH is suspended (39). In addition, events such as reduced T4, lower limb edema, and knee pain can occur; however, they have no long-term sequelae and are self-limiting following timely management. Moreover, after the side effects subside, they do not reappear after the re-initiation of therapy.

In conclusion, GHD children develop lipid metabolic disorder, and rhGH therapy can improve the blood lipid levels, as well as cIMT, in Chinese GHD children. Children treated with a high dose of rhGH exhibited more pronounced effects on growth promotion, as well as superior improvements in terms of their blood lipid levels and cIMT, but without a significant increase in adverse events. Larger longitudinal studies are now required to confirm these results and provide more evidence for the effect of rhGH replacement therapy on the cardiovascular risk in GHD children.

## Figures and Tables

**Table 1:** Comparison of height, weight, and height velocity between group A and group B before and after treatment

	Group A			Group B		
	Before treatment	After treatment	*P* value	Before treatment	After treatment	*P* value
Height (cm)	114.62±9.37	125.04±9.37	0.012	115.54±9.16	127.93±9.18	0.025
Height SDS	−2.53±0.40	−1.87±0.38	<0.001	−2.56±0.27	−1.66±0.28	<0.001
Wight (kg)	21.49±3.71	26.50±3.71	0.023	21.68±3.12	27.12±3.16	0.029
Wight SDS	−0.65±0.42	−0.25±0.44	0.016	−0.65±0.36	−0.18±0.41	0.009
BMI (kg/m^2^)	16.36±1.24	16.95±0.91	0.049	16.24±0.16	16.57±0.14	0.006
BMI SDS	0.14±0.32	0.18±0.26	0.689	0.13±0.26	0.18±0.24	0.551
HV (cm/year)	3.2±0.7	10.43±0.74	0.033	3.2±0.8	12.38±0.36*	0.024

HV, height velocity; SDS, SD score.

*P* value refers to the data before treatment vs. after treatment.

*P<0.05 compared with group A.

**Table 2:** Comparison of blood lipid levels and cIMT across the three groups

	Group A	Group B	Controls	*P*^1^	*P*^2^	*P*^3^
*Before treatment*						
TC (mmol/l)	5.07±0.19	5.10±0.18	3.78±0.24	0.541	0.016	0.018
TG (mmol/l)	1.65±0.13	1.68±0.09	0.66±0.11	0.268	0.022	0.027
LDL-C (mmol/l)	3.96±0.23	3.89±0.25	1.73±0.17	0.188	0.001	0.009
HDL-C (mmol/l)	0.75±0.08	0.71±0.08	2.23±0.15	0.163	0.012	0.009
cIMT (mm)	0.61±0.04	0.61±0.03	0.33±0.02	0.564	0.029	0.032
IGF-1 (ng/ml)	115.57±57.38	110.61±46.68	230.29±39.10	0.434	0.001	0.001
IGFBP-3 (μg/ml)	3.32±0.71	3.44±0.65	5.42±0.70	0.589	0.031	0.022
FPG (mmol/l)	5.02±0.28	5.08±0.51	5.19±0.35	0.640	0.857	0.811
						
*After treatment*						
TC (mmol/l)	4.54±0.38*	4.28±0.30*	3.79±0.24	0.056	0.032	0.029
TG (mmol/l)	1.35±0.15*	1.01±0.17*	0.67±0.10	0.039	0.026	0.031
LDL-C (mmol/l)	3.14±0.23*	2.91±0.27*	1.73±0.16	0.038	0.018	0.026
HDL-C (mmol/l)	1.23±0.09*	1.48±0.10*	2.24±0.14	0.027	0.017	0.020
cIMT (mm)	0.56±0.04*	0.52±0.04*	0.32±0.02	0.029	0.009	0.008
IGF-1 (ng/ml)	264.03±62.14*	291.93±60.54*	254.12±38.89	0.477	0.005	0.001
IGFBP-3 (μg/ml)	5.79±0.99*	6.14±0.96*	5.89±0.67	0.199	0.026	0.010
FPG (mmol/l)	5.06±0.73	5.32±0.76	5.20±0.45	0.224	0.729	0.453

cIMT, carotid intima-media thickness; FPG, fasting plasma glucose; HDL-C, high-density lipoprotein-cholesterol; IGF-1, insulin-like growth factor-1; IGFBP-3, IGF-binding protein 3; LDL-C, low-density lipoprotein-cholesterol; TC, total cholesterol; TG, triglyceride.

*P*^1^ value refers to group A vs. group B; *P*^2^ value refers to group A vs. control group; *P*^3^ value refers to group B vs. control group; level of significance: *P*<0.05; *P<0.05 compared with the data before treatment.

**Table 3:** Adverse reactions after treatment

Adverse reactions	Group A (cases)	Group B (cases)
Reduced T4	1	2
Increased fasting blood glucose	5	6
Lower limb edema	2	0
Knee pain	0	2
Totals	8	10
